# 流式细胞术在成熟B细胞非霍奇金淋巴瘤诊断和微小残留病检测中的应用中国专家共识（2025年版）

**DOI:** 10.3760/cma.j.cn121090-20241124-00476

**Published:** 2025-02

**Authors:** 中国抗癌协会血液肿瘤专业委员会 中华医学会血液学分会 中国慢性淋巴细胞白血病工作组

## Abstract

B细胞非霍奇金淋巴瘤（B-cell non-Hodgkin lymphoma，B-NHL）包含多种亚型，精准诊断依赖于多学科合作，包括病理形态学、免疫组织化学、流式细胞术（FCM）、细胞遗传学、分子生物学、影像学与临床特征等。FCM以其操作简单、快速、客观、灵敏度高和多参数分析等独特优势，在B-NHL的诊断和微小残留病（MRD）检测中发挥着不可替代的作用。随着第5版WHO造血与淋巴组织肿瘤分类和国际共识分类（ICC）的陆续发表，B-NHL中不同亚类的分型更加细化，一些分类方法和命名方式也发生了较大变化。为了提高B-NHL的诊断和治疗监测水平，规范我国对此类疾病的诊治程序，中国抗癌协会血液肿瘤专业委员会组织国内相关领域的专家经过多次讨论后，针对FCM在成熟B-NHL诊断和MRD检测中的应用，包括FCM样本来源与制备、不同亚型B-NHL免疫表型特点、结果分析与报告以及质量控制四方面制订了本专家共识。

## Abstract (en)

B-cell non-Hodgkin lymphoma（B-NHL）is made up of multiple subtypes, whose precise diagnosis relies on multidisciplinary collaboration, including pathological morphology, immunohistochemistry, flow cytometry（FCM）, cytogenetics, molecular biology, imaging and clinical features. FCM plays an irreplaceable role in the diagnosis of B-NHL and minimal/measurable residual disease（MRD）monitoring due to its unique advantages of simplicity, rapidness, objectivity, high sensitivity, and multi-parameter analysis. With the publication of the 5th edition of the World Health Organization（WHO）Classification of Haematolymphoid Tumours and the International Consensus Classification（ICC）, the classification of B-NHL has become more refined, with significant changes in some classification methods and nomenclature. To improve the diagnostic and therapeutic monitoring ability of B-NHL and standardize the diagnosis and treatment procedures of B-NHL in China, the Hematological Oncology Committee of the China Anti-Cancer Association organized relevant domestic experts to develop this expert consensus after multiple discussions. The consensus focuses on the application of FCM in the diagnosis and MRD detection of mature B-NHL including the sources and preparation of samples for FCM, the immunophenotypic characteristics of different subclasses of B-NHL, result analysis and reporting, and quality control.

B细胞非霍奇金淋巴瘤（B-cell non-Hodgkin lymphoma，B-NHL）包含多种亚型，精准诊断依赖于多学科合作，包括病理形态学、免疫组织化学、流式细胞术（flow cytometry，FCM）、细胞遗传学、分子生物学、影像学与临床特征等。FCM以其操作简单、快速、客观、灵敏度高和多参数分析等独特优势，在B-NHL的诊断和微小残留病（minimal/measurable residual disease，MRD）检测中发挥着不可替代的作用。随着第5版世界卫生组织（WHO）造血与淋巴组织肿瘤分类和国际共识分类（ICC）的陆续发表，B-NHL中不同亚类的分型更加细化，一些分类方法和命名方式也发生了较大变化[1]–[2]。为提高B-NHL的诊断和治疗监测水平，规范我国对此类疾病的诊治程序，中国抗癌协会血液肿瘤专业委员会组织国内相关领域的专家经过多次讨论后，对《流式细胞学在非霍奇金淋巴瘤诊断中的应用专家共识（2017年版）》[3]进行修订，针对成熟B-NHL部分制订了本专家共识。

一、概述

B-NHL是一组以成熟B细胞克隆性增殖为主要特征的具有高度异质性的恶性疾病，根据临床特征分为惰性B-NHL（iB-NHL）和侵袭性B-NHL（aB-NHL）两大类。iB-NHL多为中老年发病，形态上大多为小淋巴细胞，常累及外周血和骨髓，临床进展缓慢，多数呈惰性病程，可向aB-NHL转化；aB-NHL形态为中等到大，淋巴瘤细胞增殖迅速，患者的疾病进展比较快，通常发生肝、脾、淋巴结肿大及结外器官的占位性病变和骨髓累及[1]–[2]。

FCM在成熟B-NHL诊断中的作用主要包括3个方面：明确诊断、辅助诊断和MRD检测。明确诊断：主要见于具有特征表型并常以白血病形式存在的淋巴瘤类型，如慢性淋巴细胞白血病/小淋巴细胞淋巴瘤（CLL/SLL）和毛细胞白血病（HCL）；辅助诊断：主要指免疫表型不具有特异性，但具有特定遗传学或病理学特征的淋巴瘤类型，FCM作为B-NHL诊断的重要补充，起到辅助诊断的作用，常见的有：边缘区淋巴瘤（MZL）、滤泡性淋巴瘤（FL）、套细胞淋巴瘤（MCL）、淋巴浆细胞淋巴瘤/华氏巨球蛋白血症（LPL/WM）、Burkitt淋巴瘤（BL）、大B细胞淋巴瘤（LBCL）等；MRD检测：FCM在CLL/SLL的MRD检测中已得到广泛的应用和认可，在其他iB-NHL中也有一定应用价值，与分子生物学检测相互补充。

二、FCM样本来源与制备

（一）样本来源及送检要求

FCM样本来源比较丰富，包括骨髓、外周血、脑脊液、胸/腹水、心包积液和玻璃体液等浆膜腔积液以及细针穿刺等细胞学样本和来源于淋巴结、结外器官或组织的新鲜组织学样本。所有样本的处理原则为尽可能获得足够数量和活性良好的单细胞。

FCM样本的送检要求主要包括以下几点：①骨髓、外周血标本为天然单细胞悬液，抽取后置于抗凝管中（肝素或EDTA），室温保存，尽量12 h内进行处理，若放置时间超过12 h，建议选择肝素抗凝。建议第1管骨髓穿刺液送检FCM，标本量约2 ml。②体液和组织样本活性难以维持，采集后应立即送检。内镜下活检组织块推荐取长度为0.5 cm以上的组织，手术切除标本推荐送检长度1 cm以上的组织。对于纵隔或腹膜后淋巴结等深部病变，样本取材首选粗针穿刺，建议送检2条以上组织，若为细针穿刺，推荐多点位穿刺以避免漏检。③组织学样本离体后立即置于生理盐水、PBS、RPMI 1640培养液等运输介质中或用生理盐水浸湿的消毒纱布包裹后立刻送检，长途运输建议使用加入小牛或胎牛血清的RPMI 1640培养液或组织样本专用保存液，且全程保持2～8 °C低温环境，运输中可加冰袋，但需避免样本与冰袋直接接触，任何样本均不可使用含甲醛等固定剂的溶液保存或运输[4]–[8]。

（二）组织样本制备

送检FCM检测的组织学样本应紧邻形态学检查部位，组织样本送到实验室后应尽可能立即处理，使用手动研磨或匀浆仪将组织分散成单个核细胞，过滤后使用生理盐水或PBS调整细胞浓度至1×10^6^/ml～1×10^7^/ml。如标本内含有较多的结缔组织（如胃肠道、肝脏、皮肤来源样本），需预先加入消化酶消化后再进行研磨、过滤。建议在单细胞悬液制备前印片或抗体标记前制备1张涂片用于形态学观察[9]。

（三）细胞存活率检测及采集细胞数

检测前推荐采用活性染料（如7-AAD）进行细胞存活率评估。上样时采集的目的细胞总数应达到5 000个以上，特殊情况也应尽量达到1 500个。MRD检测至少需要获取2×10^5^个白细胞[10]。

三、B-NHL免疫表型特点

（一）正常B细胞免疫表型特点

正常的循环成熟B细胞表达CD19、CD20、CD22、CD79b、FMC7和膜表面免疫球蛋白（surface immunoglobulin，sIg）。成熟B细胞sIg的轻链kappa（sIgκ）和lambda（sIgλ）比值基本在1∶3和3∶1之间。在B细胞的成熟过程中，末端脱氧核苷酸转移酶（TdT）、CD34、CD10等不成熟标志物逐渐消失，胞质Ig重链重排时出现CD79a和PAX5的表达，CD20在Ig轻链重排时才开始表达，且表达强度逐步增强；CD45的表达随着B细胞的成熟逐渐增强；正常B细胞抗原的表达增强、减弱或消失多呈渐进性，而B-NHL肿瘤细胞抗原表达多为均质性增强或减弱[1]–[3]。

（二）B-NHL免疫分型抗体选择

诊断B-NHL所涉及的B细胞相关抗体，不仅应考虑免疫表型用途，还应涵盖成熟B淋巴细胞克隆性鉴定所需的Ig轻链。B-NHL表达成熟B细胞标志物（CD19、CD20、CD22和CD79b等），且限制性表达sIgκ或sIgλ（κ∶λ>3∶1或<1∶3），部分病例表现为sIgκ和sIgλ双阴性。通常先应用一线抗体确定样本中存在克隆性增生的成熟B细胞后，加做二线抗体进行精准分型。一线抗体包括：CD5、CD10、CD19、CD20、CD38、CD45、κ和λ；二线抗体包括：CD22、CD79b、FMC7、CD23、CD200、CD25、CD24、IgM、CD11C、CD43、CD103、CD81、ROR1、CD305、CD180、CD30、Bcl-2、CD138、ｃκ、ｃλ和增殖指数Ki-67等。各实验室可参考国际认可的标准化方案或专家共识［如欧洲流式联盟（Euroflow）、国际临床流式学会（ICCS）］中推荐的荧光染料和克隆号进行选择和组合[11]–[13]。

（三）iB-NHL免疫表型特点

iB-NHL主要指以成熟小B细胞克隆性增殖为特点的一组疾病，包括：单克隆B淋巴细胞增多症（MBL）、CLL/SLL、MCL、FL、LPL/WM、MZL、HCL、脾弥漫红髓小B细胞淋巴瘤（SDRPL）和伴显著核仁的脾B细胞淋巴瘤/白血病（SBLPN）。根据第五版WHO分类，其中HCL、脾边缘区淋巴瘤（SMZL）、SBLPN和SDRPL统称为脾B细胞淋巴瘤/白血病。

部分淋巴瘤具有独特的免疫学特征，结合散射光和CD5、CD10等抗原的表达特征可以对iB-NHL进一步区分，对于大部分iB-NHL，FCM结合分子和遗传学结果可以做出准确诊断（图1）[1]–[2],[14]–[21]。

**图1:**
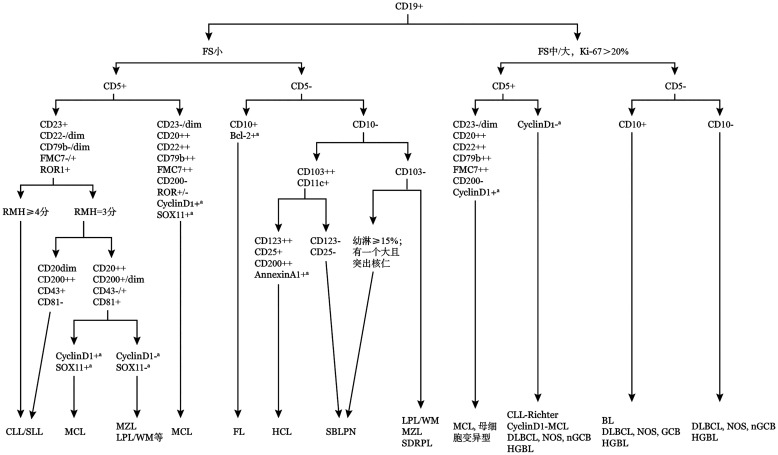
成熟B细胞非霍奇金淋巴瘤的典型免疫表型鉴别诊断流程图 **注** FS：前向散射光；CLL/SLL：慢性淋巴细胞白血病/小淋巴细胞淋巴瘤；RMH：Royal Marsden Hospital；MCL：套细胞淋巴瘤；FL：滤泡性淋巴瘤；HCL：毛细胞白血病；SBLPN：伴显著核仁的脾B细胞淋巴瘤/白血病；MZL：边缘区淋巴瘤；LPL/WM：淋巴浆细胞淋巴瘤/华氏巨球蛋白血症；SDRPL：脾弥漫红髓小B细胞淋巴瘤；DLBCL，NOS：非特指型弥漫大B细胞淋巴瘤；BL：Burkitt淋巴瘤；GCB：生发中心B细胞；nGCB：非生发中心B细胞；HGBL：高级别B细胞淋巴瘤；^a^免疫组织化学染色

1. MBL：指健康人群外周血中存在低水平单克隆B淋巴细胞（<5×10^9^/L），临床无肝/脾/淋巴结肿大、贫血、血小板减少以及淋巴瘤相关临床表现。分为3个亚型：a. 低计数CLL型MBL：CLL/SLL表型的克隆性B细胞计数<0.5×10^9^/L，且无其他iB-NHL的诊断依据；b. 高计数CLL型MBL：CLL/SLL表型的克隆性B细胞计数≥0.5×10^9^/L且总B细胞计数小于5×10^9^/L，无CLL/SLL的诊断依据；c. 非CLL型MBL：任何非CLL/SLL表型克隆性B细胞增生，无其他iB-NHL的症状或临床特征，该类型病例绝大多数起源于边缘区。

2. CLL/SLL：最常见的iB-NHL，典型的免疫表型特点为：CD19^+^、CD5^+^、CD23^+^、FMC7^-^、CD10^-^、CD200^++^、ROR1^+^、CD81^-^、CD43^+^；sIg、IgM、CD20、CD22及CD79b的表达水平低于正常B细胞（dim）。限制性表达sIgκ或sIgλ，约25％的患者CLL细胞Ig轻链为双阴性。根据CLL免疫表型RMH积分系统（CD5^+^、CD23^+^、FMC7^-^、sIg^dim^、CD22/CD79b^dim^各积1分），典型CLL积分为4～5分。积分为3分时需增加免疫组织化学（immunohistochemistry，IHC）检测CyclinD1、SOX11以及FISH检测t（11;14）等以排除MCL，同时要结合细胞学、遗传学及分子生物学检查等与MZL和LPL进行鉴别诊断，因为这些类型也可表达CD5。幼稚淋巴细胞比例大于15％时诊断为CLL伴幼稚淋巴细胞进展。

3. MCL：经典型MCL免疫表型特点：CD19^+^、CD5^+^、CD23^−/dim^、CD200^−/dim^、CD10^-^、FMC7^+^、CD81^+^、CD43^+/−^、IgM^+^；CD20、CD22、CD79b和sIg表达比CLL强；Cyclin D1^+^和SOX11^+^（IHC检测）；最主要的遗传学异常是≥95％的患者伴t（11;14）（q13;q32）从而产生IGH::CCND1融合。当患者表现为脾肿大和外周血淋巴细胞增多，但没有淋巴结疾病或血细胞减少时，称为白血病性非淋巴结型MCL（non-nodal MCL，nnMCL）。nnMCL与经典型MCL的不同之处在于：CD5阴性多见、CD200^+^、低Ki-67指数，SOX11不表达（IHC检测）。

4. FL：CD19^dim+^，CD20^+^、CD22^+^、CD79b^+^、sIg^+^，同时表达生发中心抗原CD10、Bcl-2（IHC检测）和Bcl-6（IHC检测）。因为骨髓或外周血缺乏活化的生发中心，因此这些样本中FL细胞CD10表达强度较弱或不表达，这时应依据组织病理明确诊断。该型主要的遗传学异常为由t（14;18）（q32;q21）产生的IGH::BCL2融合，见于85％～90％的FL患者。

5. LPL/WM：LPL中90％～95％为WM，超过90％的患者存在MYD88（L265P）突变。样本中常可检出单克隆性B细胞和（或）单克隆性浆细胞，B细胞免疫表型特点：CD19^+^、CD20^+^、CD22^+^、CD79b^+^、sIg^+^、CD38^+^、CD25^+^、CD27^+/−^，CD180^-^，部分患者可以CD5^+^或CD23^+^。浆细胞免疫表型特点：CD38^+^、CD138^+^、CD19^+^、CD56^-^，与正常浆细胞表型几乎没有区别，但限制性表达sIgκ或sIgλ，且轻链类型和该样本中克隆性B细胞一致。WM患者免疫球蛋白为IgM型，而LPL患者可见IgG型，一般不表达IgD。由于分泌大量Ig以及可能发生冷凝集素反应导致细胞容易粘连和聚集，从而影响检测结果，建议WM患者的样本处理（特别是sIg）在37 °C恒温水浴锅内完成。

6. MZL：累及骨髓和外周血的主要是SMZL，CD19^+^且强表达成熟B细胞相关抗原CD20、CD22、CD79b、sIg和FMC7，通常CD5^-^（5％～20％的患者可出现CD5^+^）、CD23^-^、CD10^-^、CD103^-^（少数情况下CD103可以弱阳性）、CD123^-^、CD200^−/dim^、CD43^-^、CD180^+^。CLL免疫表型RMH积分标准一般≤2分，根据CD20、CD22、CD79b、FMC7和sIg高表达以及CD5^-^和CD23^-^可与CLL鉴别；Cyclin D1^-^和CD5^-^可与MCL鉴别；CD103和Annexin A1（IHC检测）阴性可与HCL鉴别；CD10和Bcl-6（IHC检测）阴性可与FL鉴别。

7. HCL：由于细胞外呈现绒毛状凸起，在FCM检测时侧向散射光（SSC）增大，免疫表型具有特征性，因此可以直接通过FCM诊断HCL。具体表现为：CD19^+^且强表达成熟B细胞相关抗原CD20、CD22、CD79b、sIg和FMC7，特征性表达CD103和CD11c，同时伴CD123^+^、CD25^+^、CD200^++^、CD305^+^，通常不表达CD5、CD23和CD43，10％～20％的HCL病例表达CD10。HCL特异性表达Annexin A1（IHC检测），>95％的HCL患者伴BRAF（V600E）基因突变。

8. SBLPN：包括第四版WHO分类中的HCL变异型（HCL-v）、部分SMZL和部分B细胞幼淋巴细胞白血病（B-PLL）。虽然第五版WHO分类将HCL-v删除，但由于HCL-v有特征性的免疫表型（CD103^+^、CD11c^+^但CD123^-^、CD25^-^和CD200^-^，可和HCL相鉴别）和分子事件（7％～50％患者出现MAP2K1突变），为了患者个体化治疗方案的选择，建议患者具有典型HCL-v免疫表型时在报告中标注。

9. SDRPL：罕见类型，诊断依赖病理学表现（小的单形B淋巴细胞累及脾红髓），通常CD5^-^、CD10^-^，可以通过CD200^−/dim^和CD180^++^与HCL以及SMZL区分。

（四）aB-NHL免疫表型特点

包括弥漫大B细胞淋巴瘤，非特指型（DLBCL，NOS）、BL和高级别B细胞淋巴瘤（HGBL）。FCM在aB-NHL中的诊断作用有限，但依然不可或缺。对于确切亚型的精准诊断，必须结合病理、免疫组化、遗传学和临床特点。值得一提的是，当FCM检出克隆性大B细胞时，即使比例极低，结合临床侵袭性过程也应立即进行淋巴瘤的排查，尽早明确诊断，为部分患者争取治疗的时间。

aB-NHL肿瘤细胞无特征性免疫表型，通常表达泛B细胞抗原，包括CD19^+^、CD20^+^、CD22^+^、CD79a^+^和CD45^+^，CD5^+/−^，CD10^+/−^，大多数病例限制性表达sIgκ或sIgλ（双阴性也较易见）。累及骨髓的aB-NHL淋巴瘤细胞比例经常较低，混在正常B细胞中，只采用总CD19^+^细胞设门时易漏检，将前向散射光（FSC）/SSC纳入设门参数圈定大B细胞后，结合其他抗原表达可提高异常细胞检出率，获取足够数量的白细胞总数也有利于检出极低比例的大B细胞，可通过CD10是否表达协助区分生发中心（CD10^+^）和非生发中心（CD10^-^）起源。Ki-67是核抗原，FCM检测阳性判断标准要低于IHC，克隆性B细胞Ki-67的表达>20％可辅助诊断aB-NHL。对于BL和CD10^+^ DLBCL，前者表达更强的CD43和CD38，以及更高的Ki-67指数（>90％），结合形态学特点（多见蜂窝状空泡）能有效区分。约有20％ aB-NHL表达CD30，特别是EB病毒阳性患者，对于临床选择维布妥昔单抗靶向治疗有重要意义，FCM检测CD30时建议选择强荧光素标志的抗体（如PE和APC）[1]–[2],[15],[22]–[24]。

（五）复合型淋巴瘤（composite lymphoma，CL）和不一致性淋巴瘤（discordant lymphoma，DL）免疫表型特点

CL指同一解剖部位或同一包块发生2种及以上的不同组织学类型淋巴瘤，常见于一个淋巴结样本中发现如血管免疫母细胞性T细胞淋巴瘤（AITL）合并DLBCL、霍奇金淋巴瘤（HL）合并DLBCL、HL合并FL、AITL合并CLL等。DL指2个及以上解剖部位发生2种及以上不同组织学类型的淋巴瘤，多数是来源于骨髓的惰性淋巴瘤合并来源于淋巴结的侵袭性淋巴瘤，如FL合并DLBCL、CLL合并DLBCL等[25]–[26]。

（六）MRD监测

淋巴瘤患者用FCM检测MRD的样本类型受限于骨髓、外周血、脑脊液和其他浆膜腔积液，由于多数aB-NHL骨髓/外周血浸润率低，因此该方法主要适用于iB-NHL的MRD检测。为了有效减少多管检测因细胞分布不均所导致的结果偏差，建议采用八色及以上的单管抗体组合方案。CLL-MRD检测流程可参考《慢性淋巴细胞白血病微小残留病检测与临床解读中国专家共识（2023版）》[27]，抗体组合包括CD19、CD20、CD79b、CD5、CD81、CD43、ROR1和CD45。其他类型iB-NHL的FCM-MRD检测少见报道，理论上只要初发时在骨髓或外周血中能检出肿瘤细胞，并且具有淋巴瘤相关免疫表型，均可利用FCM进行MRD检测[3]。除了CLL，专家组建议其他类型淋巴瘤MRD组合方案仍应包含sIgκ和sIgλ，可在sIgκ/sIgλ/CD20/CD22/CD10/CD5/CD19/CD45八色组合方案的基础上，根据疾病类型和仪器配置适度添加1～2个标志物进行更精准组合，如MCL增加CD200，HCL增加CD103和CD11c。

检测MRD至少需要获取2×10^5^个细胞，使得方法学敏感性达10^−4^。对于追求疾病缓解最大化的临床试验、CAR-T细胞治疗、造血干细胞移植等治疗手段，选择10^−5^～10^−6^作为阈值更具临床价值，可采用二代FCM，即当获取细胞数大于2×10^6^时，敏感性能达到10^−5^以下。值得注意的是，CD20等单克隆抗体在治疗中的应用会给MRD检测技术带来假阴性结果，数据分析时应紧密结合免疫治疗史。而CAR-T细胞治疗后会导致靶点抗原的丢失和减弱，MRD检测时需要增加替代设门抗体，如CD19 CAR-T细胞治疗后，CLL可采用ROR1联合CD24作为替代设门标记，其他B-NHL可选择CD22、cCD79a、CD24^+^/CD66b^-^作为设门标记[26]–[29]。

四、结果分析与报告

CD45/SSC设门应用最为广泛，利用CD19（CD20）/SSC、CD19（CD20）/FSC或CD19（CD20）/CD45散点图，可以同时对CD19^+^CD20^+^成熟B细胞、CD19^+^CD20^−/+^或CD19^+/−^CD20^+^可疑B细胞进行设门后分析，注意CD20单抗治疗后不宜采用CD20设门。报告中除患者的个人信息、标本类型、送检日期、报告者署名及日期等一般资料外，还应详细描述淋巴瘤细胞的比例、散射光特征、系别、成熟度以及不同抗原的表达特征（增强、减弱、是否缺失以及异常获得等），散点图应同时显示正常背景细胞且避免仅对结果进行简单的描述。典型CLL可直接给出结论，其他有典型免疫表型的B-NHL可在描述免疫表型的同时，尽可能给出提示性诊断和建议（如：免疫表型符合...淋巴瘤，建议进一步检测...以明确诊断；...淋巴瘤可能性大或不排除，建议完善相关检查以明确诊断），以指导临床有方向性地完善分子遗传学和相关检测指标，尽快明确诊断。

五、质量控制

质量控制包括检验前、检验中和检验后整个过程，主要涉及样本、仪器、试剂、人员等。总体要求应遵守国家卫生健康委员会于2022年11月发布的《临床血液与体液检验技术标准》的行业标准和《白血病/淋巴瘤免疫分型检测质量控制指南》[11],[13],[30]。对于B-NHL，需特别注意以下几个方面：①用于实体瘤FCM检测的样本应与具有代表性的活检样本部位相同，以防止因采样误差而导致与形态学结果不一致；②为防止目的细胞丢失，细胞学样本不推荐使用淋巴细胞分层液分离单个核细胞；③组织学样本如红细胞较多，需加入少量肝素抗凝剂，但不可使用固定剂固定；④样本处理过程中应采用溶血剂处理红细胞，氯化铵溶血法适用于骨髓和外周血，而组织学标本尽可能使用甲酸溶血法；⑤检测Ig（细胞膜/胞质轻链或重链）时，染色前需要洗涤2～3遍，高球蛋白血症样本应在37 °C恒温水浴锅内处理；⑥sIgκ和sIgλ检测时如采用一种单抗检测结果为阴性，需更换克隆号复测，或采用多克隆抗体标志以明确为真阴性；注意部分CLL、生发中心起源DLBCL和浆膜腔积液来源样本，会出现轻链表达下调或阴性；⑦当形态学观察到疑似淋巴瘤细胞，而FCM为阴性结果时，不能排除淋巴瘤骨髓浸润，建议更换部位重新抽取骨髓，取骨髓涂片后的第1管标本再次送检。

六、小结与展望

淋巴瘤是一组高度异质性的肿瘤，FCM是诊断淋巴瘤的一种快速有效的工具。本共识综合阐述了FCM在B-NHL诊断及MRD检测中的应用，包括不同亚型B-NHL免疫表型特点、FCM检测和分析流程、质量控制和报告内容，希望能对B-NHL临床诊疗起到补充作用，为临床工作者制订治疗决策提供方向，随着FCM临床实践的进展，本共识将不断更新完善[1]–[2]。
